# Kinetic Analysis of Ex Vivo Human Blood Infection by *Leishmania*

**DOI:** 10.1371/journal.pntd.0000743

**Published:** 2010-07-13

**Authors:** Inmaculada Moreno, Mercedes Domínguez, Darío Cabañes, Carmen Aizpurua, Alfredo Toraño

**Affiliations:** Servicio de Inmunología, Centro Nacional de Microbiología, Instituto de Salud Carlos III, Majadahonda, Madrid, Spain; Institut Pasteur, France

## Abstract

The leishmanioses, vector-borne diseases caused by the trypanosomatid protozoan *Leishmania*, are transmitted to susceptible mammals by infected phlebotomine sand flies that inoculate promastigotes into hemorrhagic pools created in host skin. We assumed that promastigotes are delivered to a blood pool, and analyzed early promastigote interactions (0–5 min) with host components, which lead to parasite endocytosis by blood leukocytes, and to host infection. Promastigotes were incubated with NHS or with heparinized blood in near-physiological conditions, and we used cell radioimmunoassay and flow cytometry to measure the on-rate constants (k_+1_) of promastigote interactions with natural opsonins and erythrocytes. We obtained quantitative data for parasitized cells to determine the time-course of promastigote binding and internalization by blood leukocytes. In these reactions, promastigotes bind natural opsonins, immune adhere to erythrocytes and activate complement cytolysis, which kills ∼95% of promastigotes by 2 min post-infection. C3-promastigote binding is a key step in opsonization; nascent C3-promastigotes are the substrate for two simultaneous reactions, C3-promastigote immune adherence (IA) to erythrocytes and complement-mediated promastigote killing. The k_+1_ for IA was 75-fold greater than that for promastigote killing, showing that IA facilitates promastigote endocytosis and circumvents lysis. At 5 min post-infection, when reaction velocity is still linear and promastigote concentration is not limiting, 17.4% of granulocytes and 10.7% of monocytes had bound promastigotes, of which ∼50% and ∼25%, respectively, carried surface-bound (live) or internalized (live and dead) leishmanias. Of other leukocyte types, 8.5% of B cells bound but did not internalize promastigotes, and T cells, NK cells and CD209^+^ dendritic cells did not bind parasites. These data show that, once in contact with blood, promastigote invasion of human leukocytes is an extremely rapid and efficient reaction, and suggest that the IA reaction constitutes a central strategy for this parasite in subverting host innate immune defenses.

## Author Summary

*Leishmania* infection is transmitted to mammalian hosts by phlebotomine sand flies. During the vector's bloodmeal, promastigotes are inoculated into hemorrhagic spots in the skin or are delivered into the extracellular matrix of the dermis. In the first case, blood is involved in transmission; in the second, it apparently is not. This is important, as the cellular milieu of infection can be critical for induction of the anti-parasite immune response and the subsequent course of disease. In humans, there are few comprehensive studies of the initial stages of *Leishmania* transmission in blood. Using blood to mimic the skin hematoma, we carried out kinetic and quantitative analyses of the reaction with serum and blood components of promastigotes from two *Leishmania* species with different tropism. We describe the kinetics of the promastigote reaction pathway that leads to blood infection and provide quantitative data for the cell types infected in the first five minutes of leishmaniosis transmission.

## Introduction

The leishmanioses are a group of vector-borne zoonotic diseases caused by trypanosomatid parasites of the genus *Leishmania*. Leishmanias are heteroxenous protozoa with a life cycle in two different hosts, in the *Psychodidae* diptera of the genera *Phlebotomus* and *Lutzomyia* and in mammals. In the sand fly, *Leishmania* lives in the digestive tract as an extracellular motile flagellated promastigote; in mammals, it dwells as a sessile aflagellated amastigote inside macrophages [1].

Female phlebotomine sand flies are hematophagous arthropods that require blood proteins for oogenesis. Sand flies feed from hemorrhagic spots created in the host dermis. When feeding on a *Leishmania*-infected host, flies can ingest amastigotes or amastigote-laden macrophages and become infected. In the vector gut, amastigotes differentiate first into procyclic promastigotes and subsequently into more mature promastigote morphotypes. Promastigote differentiation generates mature, non-dividing parasites termed metacyclic promastigotes, considered the *Leishmania* forms that infect mammals [2]. In heavily infected flies, the lumen of the food canal appears choked by a promastigote-derived mucin-like gel (PSG) containing large numbers of promastigotes embedded in a filamentous proteophosphoglycan (fPPG) matrix [3]. During sand fly engorgement, PSG limits the food flow and jams the vector feeding system, hampering intake of an adequate blood meal; this prompts sand fly regurgitation and delivery of promastigotes together with saliva and fPPG to the intradermal pool, thus causing infection [4].

Leishmaniosis is transmitted to mammalian hosts when infected sand flies take a second blood meal [5], [6]. Cases of *Leishmania* infection have been reported in humans with no apparent blood uptake by the vector; in this case, promastigotes are presumably deposited into the extracellular matrix in the dermis [7]–[9]. Parasite transmission without blood involvement is also described in experimental rodent infection, in which a considerable fraction of transmitting flies apparently did not ingest blood while feeding [10], [11]; nevertheless, one of these studies shows that promastigote transmission was 2.6-fold higher among flies that had taken a second blood meal [10]. Studies of natural sand fly feeding habits showed that a majority (63–68%) of females trapped around animal shelters were blood-fed, and that 58.7% of blood-fed flies were PCR-positive for *Leishmania* DNA, double the number of positives found in non-blood-fed flies [12]. These data indicate that blood uptake by the vector is frequent in leishmaniosis transmission.

In natural *Leishmania* infection, promastigotes can therefore be delivered into hematomas or into a bloodless context in the skin. This is not an irrelevant issue, as promastigotes interact in blood with leukocyte populations, whereas in the dermal matrix they interact with fibroblasts, dermal dendritic cells (DDC), mast cells, and macrophages [13], [14]. The environment and cell target repertoire in which infection occurs can influence the course of disease development, as well as the type and intensity of immune response induced [15], [16].

Recent work in mice explored parasite fate after intradermal promastigote inoculation [13], [17], but the physiological and functional differences between the innate immune systems of mouse and man preclude direct extrapolation of results. In humans, comprehensive studies of the initial stages of leishmaniosis transmission in the blood pool are lacking, and most infection studies have been carried out using isolated leukocyte populations [18]–[26]. For a previous study, we designed an *ex vivo* model of infection in human blood to analyze the early stages of promastigote-host interaction [27]. Opsonisation, binding and internalization of promastigotes by target leukocytes occur within minutes (early infection); ensuing reactions triggered by the cells that endocytose parasites take hours or days to develop or to reach full intensity. Using two *Leishmania* species with different tropism, *L. amazonensis* and *L. donovani*, we studied the kinetics of early *Leishmania* infection of human blood, and measured the rate constants (k_+1_) of promastigote opsonization reactions and the kinetics of promastigote binding and internalization by blood leukocytes during the very early infection period (0–5 min). Based on these data, we propose a kinetic model of ex vivo human blood infection by *Leishmania* promastigotes.

## Materials and Methods

### Ethics statement

This study was approved by the Ethics Committee (Comité de Ética de la Investigación y Bienestar Animal) of the Instituto de Salud Carlos III (Ref CEI PI 12_2009). All human participants were volunteers and gave written consent.

### Parasites and cultures

*Leishmania donovani* Khartoum 1246 (MHOM/SD/43/124) and *Leishmania amazonensis* Maria (MHOM/Br/79/Maria) isolates were cultured in RPMI 1640 complete medium as described [27]. Stationary phase parasites were harvested by centrifugation (1,500×g, 15 min, 20°C), washed twice in PBS pH 7.2, and adjusted to the desired concentration.

### Promastigote labeling

Promastigotes were labeled with [^111^In]-oxine as described [27]. For labeling with 5-chloromethylfluorescein diacetate (CMFDA; Invitrogen, Carlsbad, CA), early stationary phase promastigotes in 1 ml culture medium (∼2×10^7^ cells) were incubated (15 min, 37°C) with 3 µM CMFDA; after incubation, cells were washed in PBS by centrifugation (11,000×g, 1 min) and adjusted to 10^7^ CMFDA-labeled promastigotes/ml.

### Human blood and serum collection

Blood was drawn from healthy donors into preservative-free heparin (10 IU/ml), kept at room temperature (20°C), and used in the IA reaction within hours of extraction. Normal human serum (NHS) was collected from clotted blood (20°C, 30 min) and serum aliquots stored in liquid nitrogen.

### Antibodies

Monoclonal antibodies (mAb) used were anti-CD15-PE (clone HI98), -CD14-allophycocyanin (APC) (clone M5E2), -CD3-PE-Cy5 (clone 5HIT3a) and -CD56-APC (clone B159) (all from BD Pharmingen, San Jose, CA), -CD19-APC (clone HIB19; BioLegend, San Diego, CA) and CD209-PE (clone eB-h209; eBioscience, San Diego, CA). FITC-labeled rabbit anti-human μ chain was from Dako (Glostrup, Denmark). mAb SIM 27–49 (anti-human C3 α chain; IgG2b) was Cy5-labeled (GE Healthcare) following manufacturer's instructions. Goat anti-μ (50 µg) and SIM27–49 (25 µg) were labeled with 5 µl sodium ^125^I (carrier-free, 105.36 mCi/ml; DuPont/NEN Life Science) in Iodogen (Pierce, Rockford, IL)-coated tubes and [^125^I]-SIM27–49 activity was measured [28].

### Measurement of reaction on-rate (k_+1_) constants

The *Leishmania* opsonization pathway can be depicted as a sequence of four reactions: 1) promastigote+natural antibodies→promastigote-IgM, 2)+complement→promastigote-C3 (nascent promastigote-C3 are the substrate of two subsequent competing reactions), 3) promastigote-C3+E→promastigote-E IA and 4) promastigote-C3+C5b-C9→promastigote propidium iodide (PI) uptake. In 25% NHS, the concentrations of anti-*Leishmania* IgM antibodies ([IgM_5_]), complement components and receptors for complement C3b fragments on erythrocytes ([E-CR1]) are in moderate to great excess over the concentration of promastigote IgM binding sites ([Pm_bs_]) and bound C3b with which these molecules interact in reactions (1) to (4). To simplify measurement of on-rate constants, reactions (1) to (4) were considered one-way bimolecular interactions of the type A reactant+B reactant→P (product), and analyzed as described [29], [30]. The rate of P formation is described as
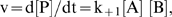
in which k_+1_ is the second-order on-rate constant for the reaction. When the concentration of reactant [B] is >> [A], [B] does not change substantially and the reaction is said to be pseudo-first-order. The velocity equation is written
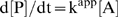
where k^app^, the apparent pseudo-first-order rate constant, is k^app^ = k_+1_ [B]. The rate expression simplifies to L (B_max_/B_max_−B_t_) = k^app^ t, in which L is the natural logarithm, B_max_ is the percent of maximum binding, and B_ti_, the percent of binding at times t_i_. Kinetic data plotted as L (B_max_/B_max_−B_ti_) against incubation time (t_i_) render a straight line of slope k^app^. The second-order rate constant of the reaction is obtained from k_+1_ = k^app^/[B]. Sigmoid kinetic data were fitted by non-linear regression using a four-parameter Hill equation (SigmaPlot 9.0).

### Promastigote-IgM binding

Aliquots (50 µl) of *L. donovani* or *L. amazonensis* promastigotes (10^7^/ml) were mixed with 50 µl 50% PBS-diluted pooled NHS and incubated for varying time periods between 0 and 60 sec. The reaction was terminated by dilution with 1 ml cold (4°C) PBS containing 2.5% FCS and 0.05% NaN_3_ (PFS buffer), followed by centrifugation (11,000×g, 1 min). To avoid cell loss, untreated promastigotes (5×10^6^) were then added to the pellet and the samples washed twice in cold PFS (11,000×g, 1 min) to remove traces of serum IgM that could block ^125^I-goat anti-μ binding. Parasites were resuspended in 0.2 ml PFS containing 2×10^5^ cpm ^125^I-goat anti-μ (10^7^ cpm/µg), incubated 1 h on ice, washed twice as above, and promastigote-bound antibody was determined.

### Promastigote-C3 deposition reaction

Assay conditions were identical to those for promastigote-IgM binding, except that the reaction was terminated by dilution with 1 ml cold PFS containing 5×10^6^ untreated promastigotes, and tube contents were washed twice by centrifugation (11,000×g, 1 min). Promastigote pellets were resuspended in 0.2 ml PFS containing 5×10^5^ cpm [^125^I]-SIM27–49 (6×10^6^ cpm/µg) and incubated 1 h on ice. After incubation, samples were processed as above and promastigote-bound [^125^I]-SIM27–49 cpm determined.

### Promastigote-erythrocyte immune adherence reaction

Aliquots (50 µl) of [^111^In]-labeled promastigotes (10^7^ cells/ml) were mixed with 50 µl heparinized blood and incubated for varying time periods (0 to 60 sec). EDTA (final concentration 5 mM) was added to terminate the reaction, and samples were immediately fractionated by centrifugation (500×g, 3 min) through 1.5 ml 72% Percoll. [^111^In]-labeled E-bound (E pellet) and free parasites (Percoll solution) were then filtered through glass fiber discs (GF/C; Whatman), washed three times with cold PBS, and retained [^111^In] cpm determined.

### Complement-mediated promastigote propidium iodide uptake

Single aliquots (50 µl) of *L. donovani* or *L. amazonensis* promastigotes (10^7^/ml) were mixed with 50 µl 50% PBS-diluted pooled NHS and incubated for varying times (0 to 140 sec). The reaction was terminated by diluting the sample with 1 ml FACSFlow sheath fluid (BD Biosciences, San José, CA) containing 5 µg/ml propidium iodide (PI; Sigma-Aldrich, St. Louis, MO) and PI uptake by killed promastigotes was measured in a FACSCalibur flow cytometer (BD Biosciences) [28].

### Determination of complement-resistant parasite numbers in stationary promastigote inoculum

To estimate the number of live parasites after opsonization in different NHS concentrations, single aliquots (0.3 ml) containing *L. amazonensis* promastigotes (2×10^5^), 10 µg/ml PI, and serially diluted (50% to 0.78%) NHS were incubated (37°C) for 1 to 9 min. *Leishmania* killing was determined by measuring promastigote PI uptake in real-time by flow cytometry (FACSCalibur). The precise percentage of complement-killed promastigotes cannot be determined by flow cytometry due to background signal from small particles and debris in stationary cultures; to reduce background, promastigotes were gated (SSC vs. FSC) and PI uptake emission measured in a dot plot of FL-2 (585/42 nm) vs. time (sec); data were analyzed with CELLQuest software (Becton Dickinson).

To measure promastigote complement-resistance in 50% NHS, aliquots (50 µl) of promastigotes (2×10^6^/ml) of six *L. amazonensis* stationary cultures (96.6% viable) were incubated in 50% pooled NHS (37°C, 5 min), after which promastigote PI uptake was measured by flow cytometry. Additional experiments (n = 3) compared PI uptake by stationary promastigotes and promastigotes enriched in metacyclic forms by centrifugation in a 10% to 30% Ficoll density gradient (“top 10” promastigotes) [31], [32]. Briefly, 1 ml of stationary-phase promastigotes (2×10^8^/ml) was layered onto a discontinuous gradient of 2 ml 10% and 2 ml 30% Ficoll solutions, and centrifuged (1300×g or 365×g, no brake; 22°C, 10 min). Promastigotes at the 10% Ficoll interface and the upper part of the 10% Ficoll cushion were pooled, diluted with RPMI 1640 complete medium and washed by centrifugation (1500×g, 15 min). The pellet, resuspended in complete RPMI 1640, was washed again and adjusted to 2×10^7^cells/ml. Aliquots (50 µl) of stationary-phase and “top 10” promastigotes were incubated (37°C, 5 min) in 50% NHS; for controls, NHS was replaced by PBS. The reaction was terminated by dilution with complete RPMI 1640 and centrifugation. Promastigote PI incorporation was determined by flow cytometry after incubating a 10 µl aliquot of each sample in 0.2 ml of PBS containing 2 µl PI (1 mg/ml). Promastigotes were gated (SSC vs. FSC) to eliminate small particles and debris, and PI uptake emission was collected in the FL2 detector through a 585/42 nm band pass filter.

After opsonization in different NHS concentrations, the percentage of live promastigotes was quantitated by light microscopy. Aliquots (50 µl) of stationary-phase *L. amazonensis* promastigotes were incubated (37°C, 5 min) in serially diluted NHS (1∶2; 50% to 0.78% concentration). The reaction was terminated by dilution with PBS, and live parasites counted under a microscope. It should be noted that after parasite incubation in ≥3% serum, motile promastigotes with a slender shape are observed very infrequently. Most parasites registered as viable undergo marked changes in cell geometry, lose the flagellum and become ellipsoidal or small round refractile bodies with limited but noticeable drifting; such changes complicate identification.

### Determination of leukocyte binding of promastigotes

To measure initial blood leukocyte binding of opsonized leishmanias, we incubated CMFDA-labeled promastigotes with heparin-treated blood and used flow cytometry to determine the percentage of each leukocyte subpopulation that bound parasites after 5 min. We mixed 1 ml CMFDA-labeled *L. amazonensis* or *L. donovani* promastigotes (10^7^/ml) with 1 ml heparinized blood, followed by incubation (37°C, 5 min, waterbath). The mixture was divided into 200 µl aliquots and E were lysed by incubation with 2 ml E-lysing reagent (EasyLyse, DakoCytomation; 10 min). Cells were washed in PBS by centrifugation (500×g, 10 min, 20°C). Each pellet was incubated (30 min, 20°C) with one of the following mAb: anti-CD14-APC, -CD15-PE, -CD3-PE-Cy5, -CD56-APC, -CD19-APC or -CD209-PE. Cells were washed in PBS by centrifugation (500×g, 10 min). Leukocyte subpopulations were identified by flow cytometry and represented in a dot plot as side scatter (SSC) vs. the specific fluorescent label of each population. The percentage of each subpopulation that bound promastigotes was analyzed in a secondary plot by independently representing the fluorescence intensity of each gated mAb-labeled population vs. that of cell-bound CMFDA-labeled promastigotes (FL1, green 530 nm) following excitation with a 488 nm argon ion laser. To account for nonspecific binding, we subtracted promastigote binding at time zero from all values registered. In heat-inactivated serum (HIS), promastigote binding by leukocytes was negligible at the times tested (Fig. S1). Use of HIS as control would require cell separation from plasma and its replacement with HIS, a less physiological approximation of infection conditions. The number of events acquired by the cytometer varied from 5×10^4^ to 2×10^5^. A total of 5×10^3^ events were counted for each leukocyte population, except in the case of CD209^+^ cells, for which 2×10^5^ leukocytes were acquired and 150 events counted.

### Time-course analysis of promastigote binding and internalization by granulocytes and monocytes

To measure the kinetics of promastigote binding and internalization by granulocytes and monocytes, 50 µl aliquots of heparinized human blood were mixed with 50 µl CMFDA-labeled *Leishmania* promastigotes (10^7^/ml) and incubated (37°C) for various times (0, 0.5, 1, 1.5, 2, 2.5, 3, 4, 5, 10, 30 and 60 min). The reaction was terminated by addition of 2 ml E-lysing reagent. After 10 min incubation, 3 ml of sheath fluid were added to each tube and centrifuged (500×g, 5 min); the pellet was washed with 5 ml sheath fluid as above and resuspended in 200 µl sheath fluid. Samples were divided in two 100 µl aliquots. To quench extracellular fluorescence, 10 µl trypan blue (TB) solution (10 mg/ml; final concentration 1 mg/ml) were added to one sample. To register the percentage of leukocytes that bound and internalized promastigotes, granulocytes and monocytes were gated by SSC vs. forward scatter (FSC) and plotted independently in a secondary plot of SSC vs. FL1 (green, 530 nm) following excitation with a 488 nm argon ion laser. Quenched samples were acquired for 10 min after TB addition. A total of 2×10^4^ events were acquired for each measurement and analyzed with CELLQuest software (BD Biosciences).

## Results

For kinetic analysis of the early mechanisms of ex vivo human blood infection by *Leishmania*, we studied promastigote interactions with NHS and with heparin-treated blood. Experiments were performed in conditions of near-physiological time (0–360 sec), temperature (37°C) and serum or blood concentrations (25% and 50%, respectively), with a constant reaction volume (0.1 ml) and promastigote inoculum (5×10^5^) throughout the study. This inoculum size corresponds to ∼5000 promastigotes delivered in a blood meal of 0.5 µl, well within the range of experimentally determined values [6], [11], [33]. All kinetic data were obtained from initial rate measurements, when reaction velocity is linear with time.

To infect blood, we used early stationary culture promastigotes taken not later than two days after they had reached maximum growth. Promastigote populations from early stationary cultures are heterogeneous; nevertheless, after contact with human blood, whose complement is cytolytic for *Leishmania*, only serum-resistant metacyclic promastigotes are alive [34]. We assume that promastigotes that survive >3 min in blood constitute the parasite population with infective capacity. Alternatively, one might use parasite inocula enriched in metacyclic promastigotes by negative selection methods, but these procedures also render heterogeneous populations [31], [35]–[38], and inoculum enrichment with metacyclic promastigotes would boost infection artificially.

To ascertain the number of complement-resistant promastigotes in the inocula, we measured PI uptake in real time by *L. amazonensis* parasites incubated with serially diluted pooled NHS. The extent of promastigote killing by human complement is dependent on serum dilution (Fig. S2). At ≥25% NHS, ∼90% of promastigotes incorporated PI very rapidly; at higher serum dilutions, promastigote PI uptake showed a gradual delay, although the percentage of promastigotes killed by the end of incubation reached maximum levels and was similar to that observed at 50% NHS. In 3% NHS and in more dilute serum conditions, most promastigotes were alive.

We used flow cytometry to assess the percentage of complement-resistant parasites after incubation in 50% pooled NHS (37°C, 5 min). Complement-mediated PI uptake by promastigotes was studied in parasites of six *L. amazonensis* stationary cultures. In three cultures, we compared PI uptake by stationary promastigotes and stationary promastigotes enriched in “top 10” metacyclic forms on a Ficoll density gradient. Promastigote aliquots were incubated in 50% NHS and PI incorporation determined by flow cytometry. Promastigotes from stationary cultures showed 4.4%±1.0% (range 2.8% to 6.8%) live parasites and controls, 96.5%±1.3%. Complement resistance was similar in stationary promastigotes and “top 10” stationary promastigotes in 50% NHS; the former showed 4.8%±1.1% and the latter, 4.6%±1.1. “Top 10” stationary promastigotes were thus not more resistant to human complement. Average recovery of “top 10” promastigotes was 16% and 35% after centrifugation at 1300×g and 365×g, respectively; this indicates that the small percentage of complement-resistant promastigotes in stationary cultures is not a function of low levels of “top 10” metacyclic forms, but that promastigotes resistant to lysis in 50% NHS are very infrequent.

To calculate the number of live promastigotes by a non-flow cytometric method, we used light microscopy. The percentage of live parasites in serum concentrations from 50% to 0.78% is indicated (Fig. S3). This method shows an average percentage of live promastigotes of 5.0%±0.7% (range 3.2% to 6.9%).

Infection of blood with promastigotes calls for the use an anti-hemostatic agent such as heparin. To determine the inhibitory capacity of heparin in *Leishmania* opsonization, we studied real-time PI uptake (i.e., killing) by *L. amazonensis* promastigotes opsonised with NHS, normal plasma, normal plasma treated with 50 µg/ml lepidurin (Refludin; Pharmion) or with heparin at concentrations of 10, 12.5, 15, 20, 40 or 80 IU/ml (Fig. S4). The velocity of promastigote PI uptake in plasma treated with 10 to 40 IU/ml heparin lagged by ∼10 sec relative to the velocity of promastigote PI uptake in NHS. To observe a ∼40-sec delay compared to NHS or to lepirudin-treated plasma, 80 IU/ml heparin was needed. Reaction intensity was identical in NHS, in lepirudin-treated plasma, and in plasma treated with different heparin concentrations; after 2 min incubation, all promastigotes had incorporated PI to the same extent. These data indicate that heparin does not interfere appreciably with parasite opsonisation in heparin-treated (10 IU/ml) human blood.

We also analyzed the influence of heparin on promastigote binding to granulocytes and monocytes in heparin-treated (10 IU/ml) human blood. Binding of *L. amazonensis* promastigotes by granulocytes and monocytes after 1, 3, and 5 min incubation in heparin-treated blood was indistinguishable from that in lepidurin-treated (50 µg/ml) blood (Fig. S5). At a 10 IU/ml concentration, heparin does not affect the course of human blood infection by *Leishmania*.

### Kinetics of natural anti-*Leishmania* IgM binding to promastigotes

**Figure 1:**
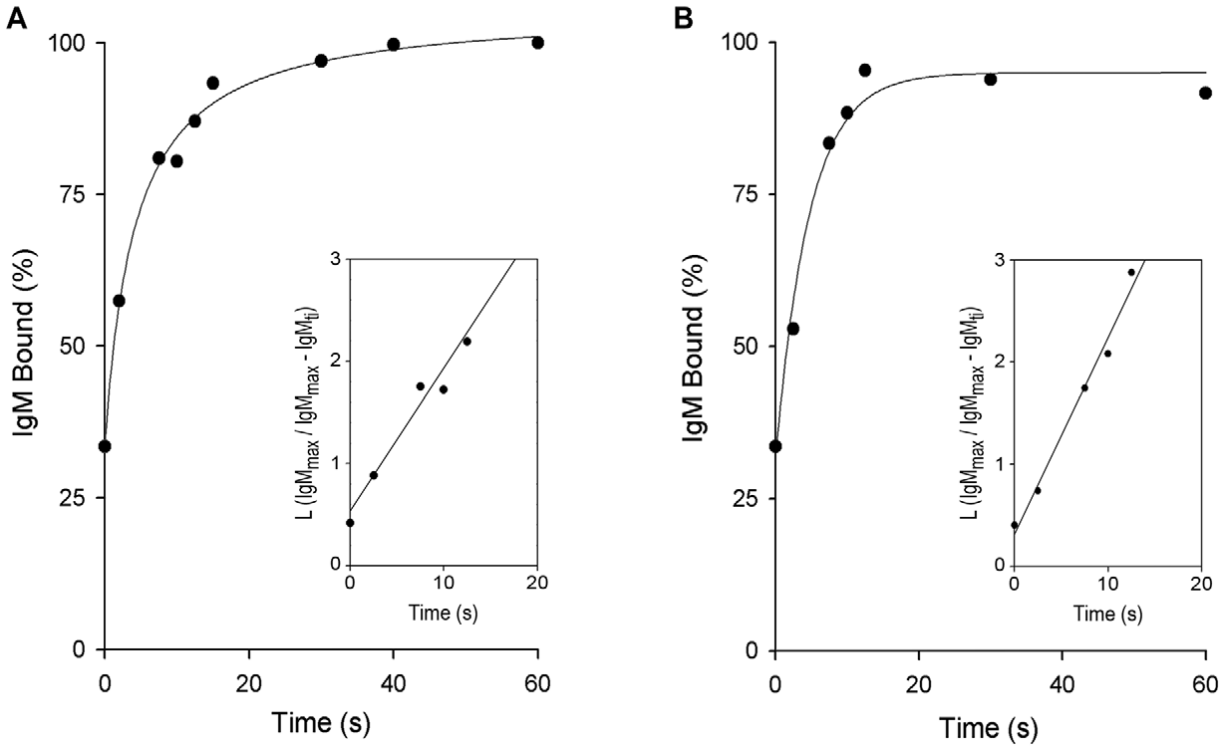
Time course of normal serum IgM binding to *L. donovani* and *L. amazonensis* promastigotes. Aliquots (50 µl) of *L. donovani* or *L. amazonensis* promastigotes (10^7^/ml) were mixed with 50 µl 50% NHS and incubated (37°C) for varying times. Promastigotes were then washed by centrifugation (11,000×g, 1 min) with cold PFS. Untreated promastigotes (5×10^6^) were added to the pellet, washed twice in cold PFS, and promastigote-bound IgM measured with ^125^I-goat anti-μ antibody. Each point (mean of triplicate samples) is expressed as a percentage of the point of maximum IgM binding in one representative experiment of five performed for each species. (A) *L. donovani*, (B) *L. amazonensis*. Insets: plots of L (IgM_5max_/IgM_5max_−IgM_5ti_) against reaction time (t_i_), from which k^app^ values were derived.

Non-immune serum from most vertebrates contains natural anti-trypanosomatid antibodies that act as an innate recognition system in the host [28], [39]. To determine the velocity of IgM binding to *Leishmania* parasites, we measured the association rate constant (k_+1_) by incubating promastigotes in NHS for varying times. The reaction was terminated by sample dilution with PFS, and promastigote-bound IgM was measured using ^125^I-goat anti-μ. Pentameric IgM (IgM_5_) binding reached maximum after 20 sec incubation for both *L. donovani* (Fig. 1A) and *L. amazonensis* (Fig. 1B). [IgM_5_] in adult NHS is ∼1.3 g/L; if the M_r_ of IgM_5_ is considered to be 950,000, then serum [IgM_5_] is ∼1.4×10^−6^ M. Exhaustive adsorption of 25% NHS with *L. amazonensis* and *L. donovani* promastigotes removed ∼15% and ∼30% of IgM_5_, respectively [28], indicating that [IgM_5_] anti-*L. amazonensis* in unadsorbed serum is ∼0.5×10^−7^ M and that of anti-*L. donovani*, ∼1×10^−7^ M. At equilibrium, *L. amazonensis* and *L. donovani* promastigotes bind ∼5,000 and ∼2,500 IgM molecules/cell, respectively. Early in the promastigote-IgM_5_ interaction, antibody binding is assumed to be monovalent [29], which would render [Pm_bs_] on *L. amazonensis* promastigotes of ∼4.1×10^−11^ M and on *L. donovani* promastigotes of ∼2.1×10^−11^ M. In a monovalent promastigote-IgM_5_ interaction, the [IgM_5_]/[Pm_bs_] ratio is ∼1,300∶1 in *L. amazonensis* and ∼5,000∶1 in *L. donovani*; in the case of a decavalent promastigote-IgM_5_ interaction, this ratio would be ∼130∶1 (*L. amazonensis*) and ∼500∶1 (*L. donovani*). In both cases, the promastigote-IgM_5_ interaction obeys pseudo-first-order kinetics. The percentage of IgM_5_ bound, plotted as L (IgM_5max_/IgM_5max_−IgM_5ti_) against reaction time (t_i_), gives straight lines whose slopes are the k^app^ values of the reactions, ∼0.23 sec^−1^ for *L. amazonensis* and ∼0.18 sec^−1^ for *L. donovani*. Second-order rate constants were obtained from k_+1_ = k^app^/[IgM_5_], with values of ∼4.4×10^6^ M^−1^ sec^−1^±0.05×10^6^ M^−1^ sec^−1^ for *L. amazonensis* and ∼1.8×10^6^ M^−1^ sec^−1^±0.05×10^6^ M^−1^ sec^−1^ for *L. donovani*. The mean k_+1_ for natural IgM_5_ binding to promastigotes of both *Leishmania* species was ∼3×10^6^ M^−1^ sec^−1^.

### Time course of C3 deposition on *Leishmania* promastigotes

Early in *Leishmania* infection, human complement has effects both advantageous for and harmful to the promastigote. Complement activation triggers binding of C3 fragments to the parasite, which mediates IA and *Leishmania* internalization by host leukocytes; however, promastigote-C3 binding also nucleates assembly of the C5 convertases, activating the complement lytic cascade that kills the parasites.

**Figure 2 pntd-0000743-g002:**
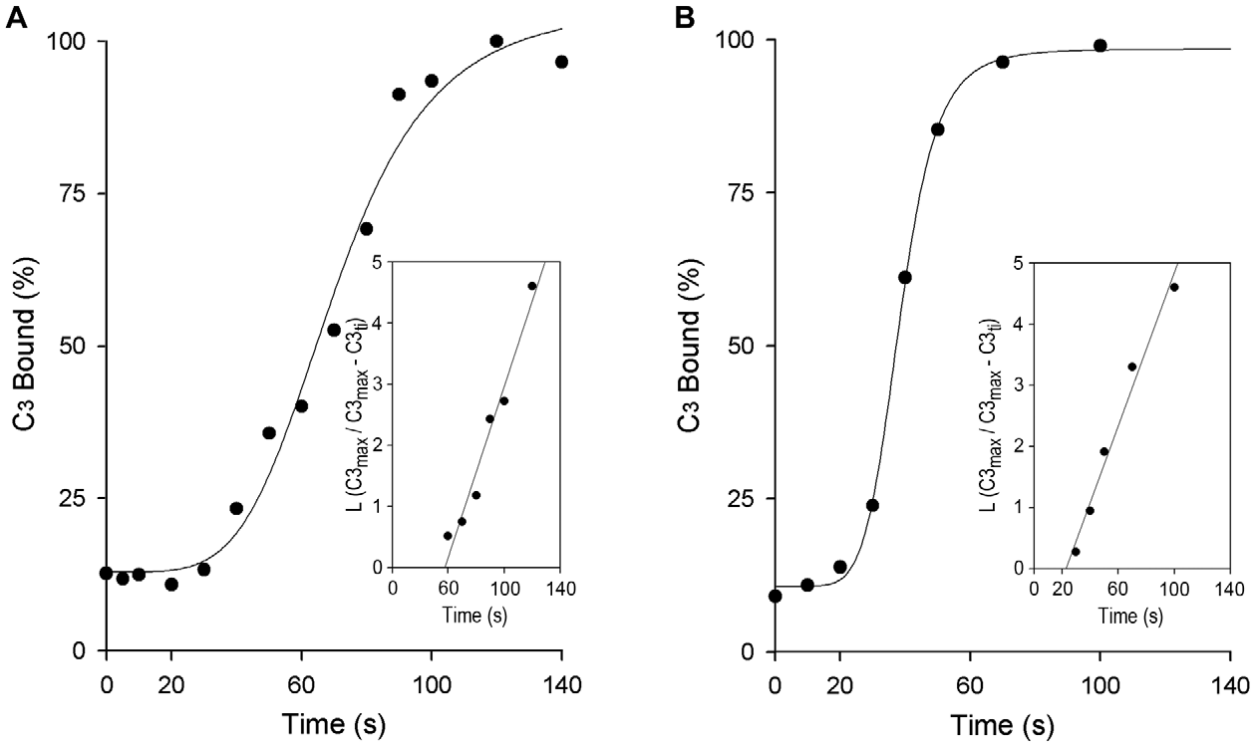
Kinetics of C3 deposition on *L. donovani* and *L. amazonensis* promastigotes. Duplicate aliquots (50 µl) of promastigotes (10^7^/ml) were mixed with 50 µl 50% NHS and incubated (37°C) for the times indicated. Promastigotes were then washed twice by centrifugation (11,000×g, 1 min) in cold PFS, and promastigote-bound C3 measured with ^125^I-SIM27–49 (anti-human C3α mAb). C3 binding (mean of duplicate values) from one representative experiment. (A) *L. donovani* (*n* = 4), (B) *L. amazonensis* (*n* = 5). Insets: plots of L (C3_max_/C3_max_−C3_ti_) against time (t_i_), from which k^app^ constants of C3 deposition reactions were obtained.

To establish how *Leishmania* circumvents complement effector activity, the kinetics and velocity of complement activation must be understood. We incubated promastigotes with NHS and measured the reaction time course and k_+1_ of promastigote-C3 binding. Promastigote-C3 binding follows a sigmoidal course, with an initial ∼30-sec lag during which 10 to 20% of total C3 ligands are fixed; C3 molecules that bind during this lag period probably attach to IgM. This amount of C3 is insufficient to activate the alternative pathway and the lytic cascade, but is sufficient to establish multipoint contacts with clustered E-CR1, triggering the IA reaction [40]–[42]. Thereafter, C3 binds at an exponential rate for ∼1 min, during which C3 is bound at an average rate of ∼1,800 molecules/sec for *L. donovani* (Fig. 2A) and ∼1,200 molecules/sec for *L. amazonensis* (Fig. 2B). During the C3 lag period, IgM_5_ binding to *Leishmania* reaches maximum and the [IgM_5_] bound is ∼4.1×10^−11^ M (*L. amazonensis*) and ∼2.1×10^−11^ M (*L. donovani*).

In 25% NHS, the concentration of complement classical pathway components ([C1–C3]) ranges from 4.5×10^−8^ M for C1 to 1.75×10^−6^ M for C3. If the C1 interaction with promastigote-bound IgM_5_ is considered the rate-limiting step in classical complement pathway activation, the serum C1 concentration is ∼2000-fold greater than that of promastigote-bound IgM_5_ for *L. amazonensis* and ∼1000-fold for *L. donovani*. [C1–C3] is >> [promastigote-IgM_5_], and C3 binding to promastigotes would proceed under pseudo-first order conditions. We analyzed the late phase of the exponential course, fitting the data to a pseudo-first order rate equation. Plots of the percentage of C3 bound as the L (C3_max_/C3_max_−C3_ti_) against time (t_i_) yielded k^app^ of ∼0.063 sec^−1^ for *L. amazonesis* and ∼0.061 sec^−1^ for *L. donovani*. The second-order rate constants were ∼3.6×10^4^ M^−1^ sec^−1^±0.01×10^4^ M^−1^ sec^−1^ for *L. amazonesis* and ∼3.5×10^4^ M^−1^ sec^−1^±0.005×10^4^ M^−1^ sec^−1^ for *L. donovani*; the mean k_+1_ value for *Leishmania* promastigote-C3 opsonization was ∼3.5×10^4^ M^−1^ sec^−1^.

### Promastigote-erythrocyte immune adherence reaction

In human blood, C3-opsonized promastigotes immune adhere to erythrocytes [27]. IA is a C3-mediated innate immune mechanism, almost ubiquitous in mammals, that enhances phagocytosis and clearance of opsonized microorganisms from blood [43]. To study the velocity of IA in the reaction sequence of *Leishmania* infection, we followed the kinetics of IA complex formation and determined the on-rate constant of the interaction between promastigote-C3 and erythrocytes.

**Figure 3 pntd-0000743-g003:**
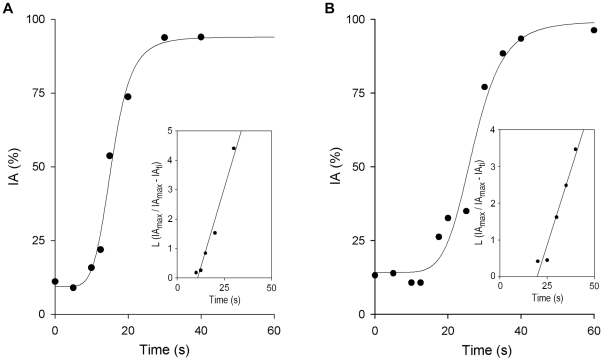
Kinetics of the *Leishmania* immune adherence (IA) reaction in human blood. Aliquots (50 µl) of [^111^In]-labeled promastigotes (10^7^cells/ml) were mixed with 50 µl heparinized blood and incubated (37°C) for the times indicated. Samples were then fractionated by centrifugation (500×g/3 min) through 72% Percoll; free and erythrocyte-bound parasites were collected separately and [^111^In] cpm determined in each fraction. The IA kinetic profile is calculated as [^111^In]-promastigote cpm bound to erythrocytes relative to total [^111^In]-promastigote cpm at each time point, and expressed as a percentage of maximum binding of triplicate samples from one representative experiment. (A) *L. donovani* (*n* = 6), (B) *L. amazonensis* (*n* = 6). Insets: plots of L (IA_max_/IA_max_−IA_ti_) against incubation time (t_i_), from which k^app^ values for *Leishmania* IA reaction were obtained. Blood from three different donors was used and two experiments were performed for each blood sample.

Within seconds of serum contact, nascent C3-opsonized promastigotes bind to CR1 on E and form promastigote-E IA complexes. This interaction has an initial lag time of ∼15 sec followed by a period in which IA complexes are formed at an exponential rate until the reaction reaches completion at 30 to 40 sec of incubation. The IA reaction is so rapid that it gives the impression that promastigote-E binding proceeds before C3 opsonization (Fig. 3A, B). Considering the average number of CR1 molecules per erythrocyte to be 500 [44], the [E-CR1] in this assay is ∼2.1×10^−9^ M. At equilibrium, *L. donovani* promastigotes bind ∼180,000 C3 molecules/cell [28]; at the onset of the IA reaction in *L. donovani* (∼12 sec), there are ∼27,000 C3 molecules (∼15% of maximum binding) bound to promastigotes, indicating a promastigote-bound C3 concentration ([Pm-C3]) of ∼2.2×10^−10^ M. At the onset of the IA reaction, the [E-CR1]:[Pm-C3] ratio is thus ∼10. In the case of *L. amazonensis*, at equilibrium promastigotes bind ∼120,000 C3 molecules/cell, and at the onset of the IA reaction (∼15 sec) there are ∼22,000 C3 molecules bound/cell (∼18% of maximum binding); this yields a [Pm-C3] of ∼1.8×10^−10^ M, and a [E-CR1]:[Pm-C3] ratio of ∼12. In these assays, the [E-CR1] exceeds the [Pm-C3] by ∼10-fold and the reaction proceeds under pseudo-first-order conditions. Regression analysis of the percentage of IA plotted as L (IA_max_/IA_max_−IA_ti_) against incubation time (t_i_) yielded k^app^ values of ∼0.15 sec^−1^ (*L. amazonensis*) and ∼0.21 sec^−1^ (*L. donovani*), and k_+1_ constants of ∼0.8×10^8^ M^−1^ sec^−1^±0.02×10^8^ M^−1^ sec^−1^ (*L. amazonensis*) and ∼1×10^8^ M^−1^ sec^−1^±0.04×10^8^ M^−1^ sec^−1^ (*L. donovani*). The average k_+1_ for the *Leishmania* IA reaction was thus ∼9×10^7^ M^−1^ sec^−1^±0.4×10^7^ M^−1^ s^−1^.

### Complement-mediated PI uptake by promastigotes

**Figure 4:**
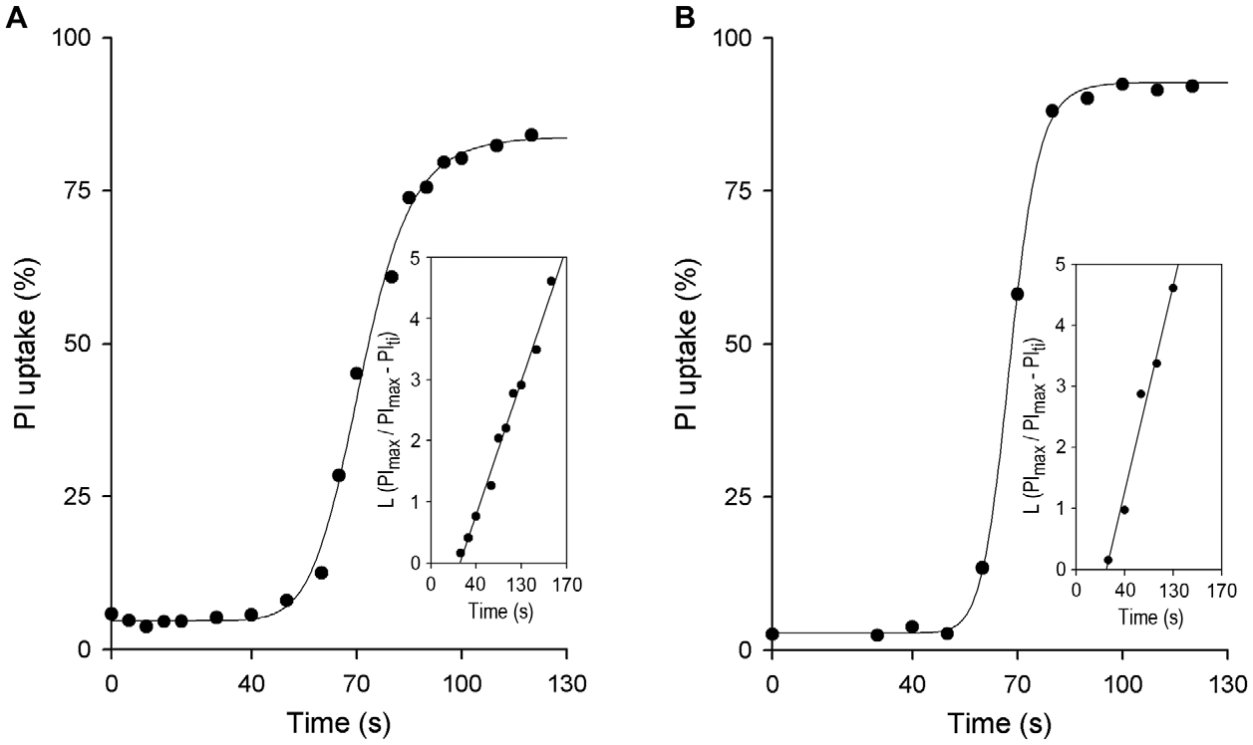
Kinetics of promastigote propidium iodide (PI) uptake in NHS. For each time point, single aliquots (50 µl) of promastigotes (10^7^cells/ml) were mixed with 50 µl 50% pooled NHS and incubated (37°C) for the times indicated. Samples were then transferred into 1 ml PBS containing PI (5 µg/ml), and promastigote membrane damage registered as PI uptake in a flow cytometer. The time course of promastigote PI uptake is shown for a representative experiment. (A) *L. donovani* (*n* = 5), and (B) *L. amazonensis* (*n* = 4). Insets: plots of L (PI_max_/PI_max_−PI_ti_) against incubation time (t_i_), from which k^app^ values were derived for promastigote PI uptake reactions.

Complement activation leads to assembly of the C5 convertases (C4b3b2a, C3b_2_Bb), triggering the cytolytic complex (C5b–C9) that causes promastigote death. We measured the time course of parasite membrane damage by complement as PI uptake and determined the on-rate constant of the reaction. The kinetics of promastigote PI uptake is very rapid; it begins at ∼50 sec after complement activation and by ∼100 sec after serum contact, most leishmanias have incorporated PI (Fig. 4A, B). During that period, the [Pm-C3] varies from ∼6×10^−10^ to ∼1×10^−9^ M in *L. amazonensis* and from ∼5×10^−10^ to ∼1.5×10^−9^ M in *L. donovani*.

In 25% human serum, the concentration of C5b–C9 components ranges from 9.2×10^−8^ M for C5 to 2.1×10^−7^ M for C9. C5b deposition is considered the rate-limiting step; at the onset of the lytic reaction, [C5b–C9] is greater than [promastigote-C3] by ∼150-fold (*L. amazonensis*) and ∼180-fold (*L. donovani*). The rate of complement-mediated PI uptake by promastigotes was calculated by plotting the percentage of PI incorporation as L (PI_max_/PI_max_−PI_ti_) against incubation time (t_i_). k^app^ values for *L. amazonensis* and *L. donovani* PI uptake were 0.13 sec^−1^ and 0.083 sec^−1^, respectively, and the k_+1_ constants were ∼1.4×10^6^ M^−1^ sec^−1^±0.008×10^6^ M^−1^ sec^−1^ (*L. amazonensis*) and ∼0.9×10^6^ M^−1^ sec^−1^±0.005×10^6^ M^−1^ sec^−1^ (*L. donovani*). The average k_+1_ value for promastigote PI uptake was ∼1.2×10^6^ M^−1^ sec^−1^.

The on-rate constant values of opsonization, immune adherence, and PI uptake reactions of *L. donovani* and *L. amazonensis* promastigotes in human blood are summarized in Table 1.

**Table 1:** On-rate constants of opsonization and immune adherence reactions of *L. donovani* and *L. amazonensis* promastigotes in human blood.

	*L. donovani*		*L. amazonensis*		
Reaction	k^app^ (s^−1^)	k_+1_ (M^−1^s^−1^)	k^app^ (s^−1^)	k_+1_ (M^−1^s^−1^)	∼k_+1_ a)
Pm+IgM_5_→Pm−IgM_5_	0.180	1.8×10^6^±0.050×10^6^	0.230	4.4×10^6^±0.050×10^6^	3.0×10^6^
Pm−IgM_5_+C3→Pm−C3	0.061	3.5×10^4^±0.005×10^4^	0.063	3.6×10^4^±0.010×10^4^	3.5×10^4^
Pm-C3+E→Pm-E	0.210	1.0×10^8^±0.040×10^8^	0.150	0.8×10^8^±0.020×10^8^	9.0×10^7^
Pm-C3+C5b-C9→Pm Lysis	0.083	0.9×10^6^±0.005×10^6^	0.130	1.4×10^6^±0.008×10^6^	1.2×10^6^

a Mean k_+1_ constant for *L. amazonensis* and *L. donovani* promastigotes.

### Leukocyte subpopulations that bind promastigotes in early infection in blood

**Figure 5:**
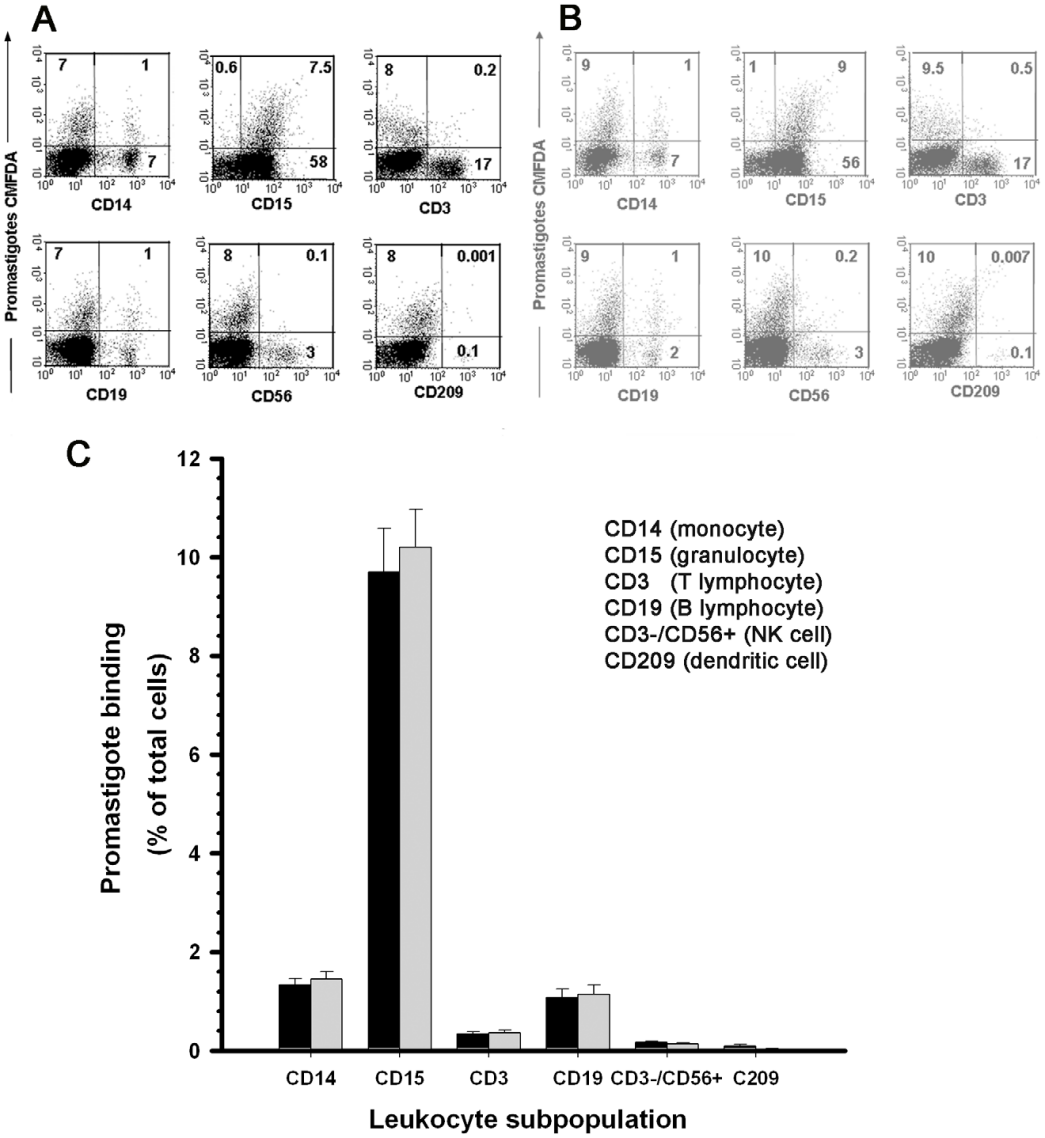
Blood leukocyte subpopulations that bind promastigotes in early infection. CMFDA-labeled promastigotes were incubated (5 min) with heparinized blood and the percentage of each *Leishmania*-binding leukocyte subpopulation was measured by flow cytometry. Leukocyte subpopulations were stained with fluorochrome-labeled mAb and identified in a dot plot as side scatter (SSC) vs. the specific fluorescent label. The percentage of each promastigote-binding subpopulation was analyzed in a secondary plot representing the fluorescence intensity of each gated mAb-labeled population vs. that of cell-bound CMFDA-labeled promastigotes. Dot plots of a representative experiment show cells in the gated populations that bound CMFDA-labeled (A) *L. amazonensis* or (B) *L. donovani* promastigotes. (C) Cells of each subpopulation that bound promastigotes expressed as a percentage of total leukocytes in the sample. Results are shown as the percentage (mean ± SEM) of 14 experiments performed with blood of six donors. (▪) *L. amazonesis*, (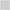) *L. donovani* promastigotes.

To measure initial blood leukocyte binding of opsonized leishmanias, we incubated CMFDA-labeled promastigotes with heparin-treated blood and used flow cytometry to determine the percentage of each leukocyte subpopulation that bound parasites after 5 min. Leukocyte subpopulations were identified with fluorochrome-labeled anti-CD15 (for granulocytes), -CD14 (monocytes), -CD3 (T cells), -CD19 (B cells), -CD56^+^ (NK cells) and -CD209 (monocyte/dendritic cells) mAb. Data from a representative experiment are shown (Fig. 5) in which fluorescence intensity of each gated leukocyte subpopulation is represented in a secondary plot against that of cell-bound CMFDA-labeled promastigotes (FL-1) as the percentage of each subpopulation that bound *L. amazonensis* (Fig. 5A) or *L. donovani* (Fig. 5B) parasites. Cells of each subpopulation that bound promastigotes are expressed as a percentage of total leukocytes in the sample (Fig. 5C; mean for eight experiments). After 5 min incubation, 13% of leukocytes bound promastigotes, of which 10.7%±0.15% were CD14^+^, 76.3%±0.8% CD15^+^, 2.7%±0.1% CD3^+^, 8.5%±0.2% CD19^+^, 1.3%±0.02% CD3^−^ CD56^+^ and 0.49%±0.03% CD209^+^ cells. A substantial fraction of B cells (8.5%) bound promastigotes in this early period. Granulocytes are the main subpopulation that bound promastigotes; nevertheless, the percentage of promastigote-binding cells in each subpopulation was nearly identical for CD15^+^ (19.1±2.4%), CD14^+^ (17.3%±2.3%) and CD19^+^ (17.4%±2.5%) (Fig. S6). Other leukocyte subpopulations did not bind promastigotes appreciably.

### Time-course of granulocyte and monocyte binding to and internalization of promastigotes in human blood

We infected heparin-treated blood with CMFDA-labeled *L. amazonensis* or *L. donovani* promastigotes. At various times post-infection, granulocytes and monocytes were gated by SSC vs. FSC, and the percentage of each subpopulation that bound parasites was calculated in a secondary dot plot by representing SSC-gated cells against fluorescence intensity of granulocyte- and monocyte-bound CMFDA-labeled promastigotes (FL1). We simultaneously determined the percentage of granulocytes and monocytes that internalized promastigotes by quenching fluorescence emitted by cell-bound complement-killed CMFDA-labeled promastigotes, using trypan blue [45].

**Figure 6:**
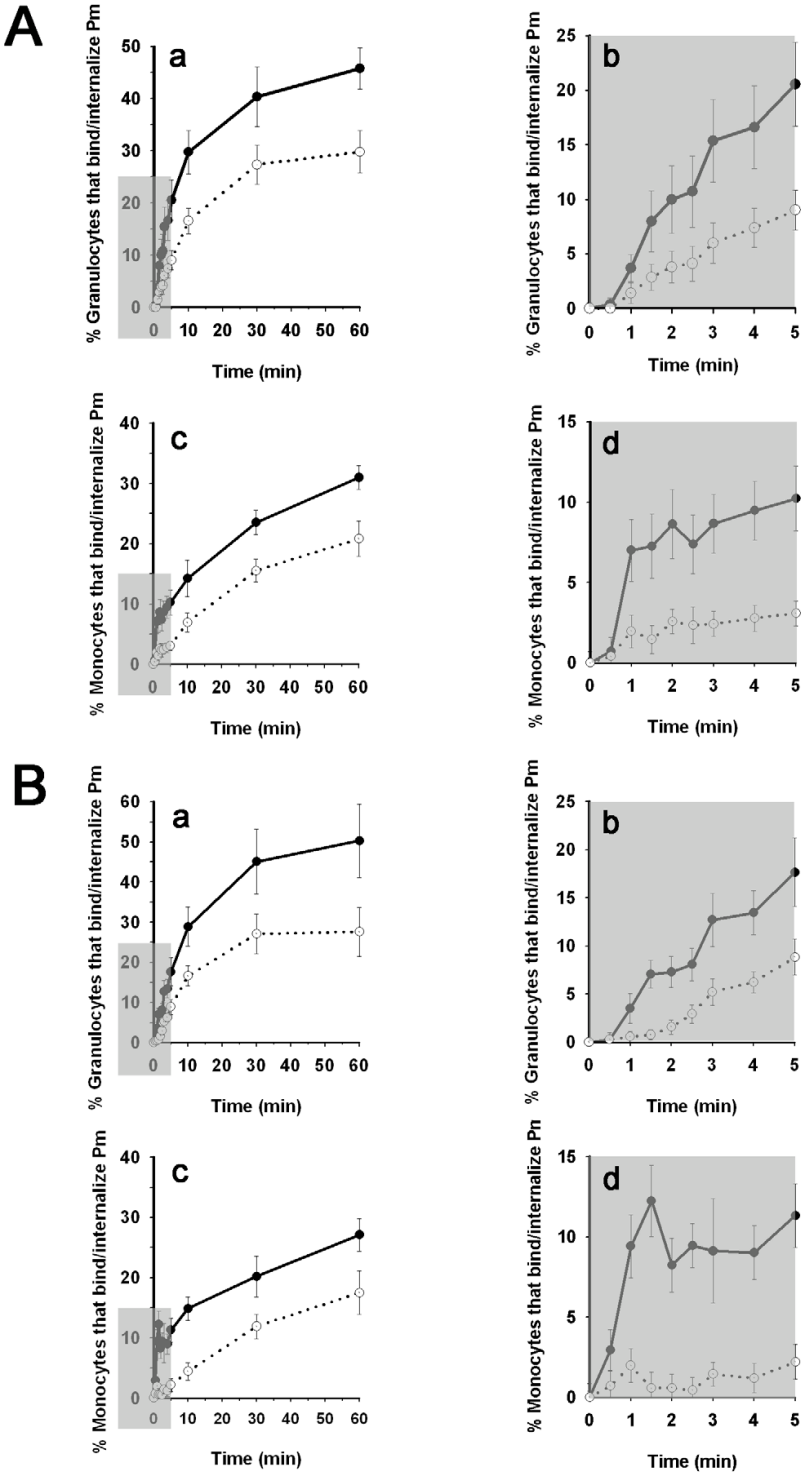
Kinetics of leukocyte binding and internalization of *L. amazonensis* and *L. donovani* promastigotes in human blood. Aliquots of heparinized human blood were infected with CMFDA-labeled *L. amazonensis* or *L. donovani* promastigotes and incubated (37°C) for various times (0–60 min). Samples were then processed as indicated in Methods. To calculate the percentage of granulocytes and monocytes that bound and internalized parasites, cells were gated by SSC vs. FSC and plotted independently in a secondary plot of SSC vs. FL1 (green, 530 nm). The percentage of cells that internalized promastigotes was determined using trypan blue to quench the extracellular fluorescence emitted by cell-bound complement-killed CMFDA-labeled promastigotes. (A) For *L. amazonensis* promastigotes: (a) Time course of granulocyte binding and internalization (•) or internalization (○); (b) data from inset (shaded area) in (a); (c) time course of monocyte binding and internalization (•) or internalization (○); (d) data from inset (shaded area) in (c). (B) Data as above (a–d) are shown for *L. donovani*. Results from seven (*L. donovani*) and ten (*L. amazonensis*) experiments with blood from six donors.

The time course of granulocyte and monocyte binding of promastigotes was linear in the first 5 to 10 min of infection. Promastigote concentration subsequently became limiting and the kinetics followed a hyperbolic course; the binding reaction was complete by 60 min (Fig. 6). After 30 min incubation, 39.2% of granulocytes (∼94% of total binding) had already bound *L. amazonensis* and 45.1% (∼90% of total binding) had bound *L. donovani* promastigotes. At this time, 23.5% of monocytes (∼76% of total binding) had bound *L. amazonensis* and 20.2% (∼75% of total binding), *L. donovani* promastigotes. We analyzed leukocyte binding and promastigote internalization in the period between 0 and 5 min, when the velocity of promastigote binding is proportional with time and the cytolytic activity of complement on *Leishmania* is probably not yet complete (Fig. 6A,B; insets). After 3 min incubation, the ratio of cells that bound∶internalized promastigotes was 12.9∶5.4% for granulocytes and 8.9∶2% for monocytes (mean value of *L. amazonensis* and *L. donovani*); after 5 min, these values were 17.4∶8.4% for granulocytes and 10.7∶2.6% for monocytes.

## Discussion

**Figure 7:**
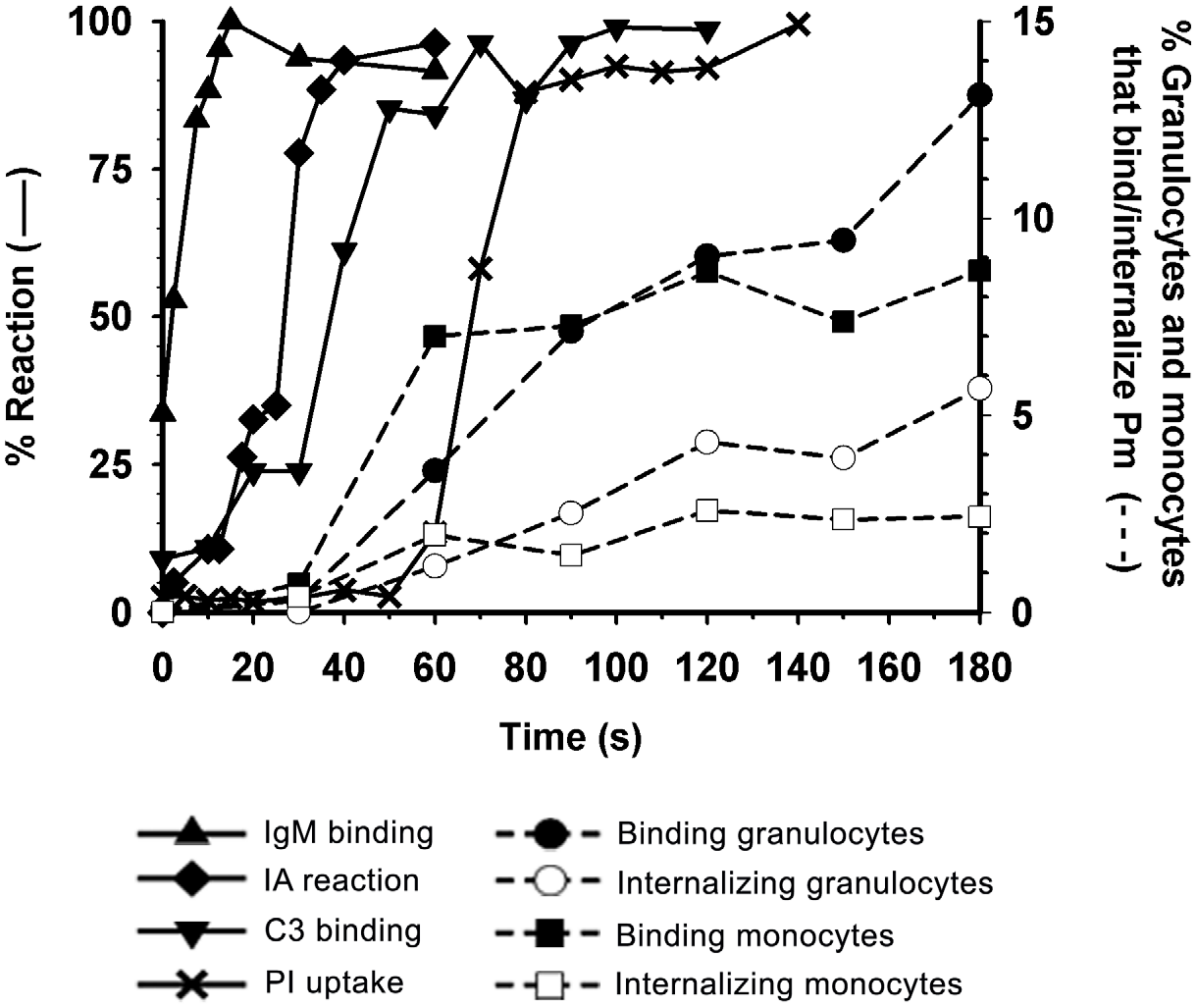
Kinetics of *L. amazonensis* promastigote interactions with host components during early infection in blood. Kinetic data are taken from the promastigote opsonization reactions shown in Figs. 1 [Fig pntd-0000743-g002] [Fig pntd-0000743-g003] to 4, and from the granulocyte and monocyte binding and internalization reactions in Fig. 6. Promastigote-IgM binding (▴), promastigote IA reaction (♦), promastigote-C3 binding (▾), promastigote PI uptake (**X**), granulocytes that bind and internalize promastigotes (•), granulocytes that internalize promastigotes (○), monocytes that bind and internalize promastigotes (▪) and monocytes that internalize promastigotes (□).

Phlebotomine sand flies transmit promastigotes to mammalian hosts in two ways, by direct inoculation of parasites into a blood pool in the skin or by their delivery into the interstitial space of the dermis [4], [14]. Here we used human blood as a surrogate model of skin hematoma for a comprehensive kinetic analysis of promastigote interactions with host blood components in the first minutes of infection. As the time course of *L. amazonensis* and *L. donovani* promastigote reactions were similar, we compiled only the data for *L. amazonensis* (Fig. 7).

Immediately after promastigote interaction with human blood, natural anti-trypanosomatid IgM/IgG antibodies bound the parasite with a hyperbolic course characteristic of first-order or pseudo-first order reactions; in contrast, the kinetics of promastigote-C3 deposition, promastigote IA, and promastigote PI uptake showed S-shaped curves, indicating cooperative (IA) or multimolecular (C3 deposition, PI uptake) mechanisms. Once opsonization was triggered, all reactions proceeded simultaneously, and by ∼2 min most parasites had been killed by complement activity. In human blood, *Leishmania* survival probably depends on the relative rate by which newly C3-opsonized promastigotes are incorporated into IA and PI uptake reactions. The 75-fold greater velocity constant of the promastigote IA (k_+1, Pm IA_∼9×10^7^ M^−1^ sec^−1^) than that of promastigote PI uptake (k_+1, Pm PI_∼1.2×10^6^ M^−1^ sec^−1^) permitted the IA reaction to be 50% complete by ∼30 sec before parasite PI uptake began. PI incorporation into promastigotes started ∼50 sec after serum contact, and required an additional 20 sec to reach 90% maximum; due to these differences in reaction times, complement killing begins at ∼70 sec after serum contact. In this interval, *Leishmania* IA can ferry promastigotes to blood leukocytes.

Leukocyte populations that express receptors for opsonic ligands, principally C3 complement fragments, bound promastigotes in proportion to the concentration of each subpopulation in the blood pool. In human blood, granulocyte concentration exceeds that of monocytes, B cells and NK cells by 6- to 8-fold, and that of natural killer (NK) and CD209^+^ dendritic cells (DC-SIGN) by two to three orders of magnitude; the latter constitute a very small cell population (0.01–0.05%) that binds diverse microorganisms *in vitro*, including promastigotes and axenic amastigotes of *Leishmania* [46]–[48].

At 5 min post-infection, 13% of blood leukocytes bound promastigotes in the proportion granulocytes (76.3%)>monocytes (10.7%)>B lymphocytes (8.5)>CD3^+^ cells (2.7%)>CD3^−^CD56^+^ cells (1.2%) cells>CD-209^+^ cells (0.5%) (%Fig. 5C). This shows that CD3^+^, NK and DC-SIGN^+^ cells have no relevant role in early blood infection. In addition to granulocytes and monocytes, ∼8.5% of B lymphocytes bound promastigotes; to our knowledge, this interaction has not been previously reported. B cell binding of opsonized promastigotes was confirmed with Raji lymphoblastoid B cells, which bound C3-opsonized promastigotes to the same extent as U937 monocytes (unpublished data). Promastigote-C3 binding by B cells is probably mediated by the IA reaction. In autologous serum and in PBS/RPMI 1640 medium, human erythrocytes transfer CR1-bound C3-opsonized promastigotes or antigen-antibody immune complexes (IC) to granulocytes, monocytes, B lymphocytes, and U937 cells [27], [49]–[51]. Binding of C3-opsonized promastigotes allows B lymphocytes to present antigens to monocytes and macrophages through a CR2-mediated reaction similar to the transfer of E-bound C3-IC to B cells [52]. B lymphocytes have a still-undefined role in host immune response to *Leishmania*. Early data showed that BALB/c mice depleted of B cells by anti-IgM treatment had enhanced resistance to *L. major* infection [53] and subsequent studies indicated that B lymphocytes are involved in mouse susceptibility to *Leishmania* infection and disease pathogenesis [54]–[57]. Our results confirm that B lymphocytes have an early role in *Leishmania* immunity.

In the ex vivo *Leishmania* blood infection experiments, promastigote inoculum was 5×10^3^ parasites/0.5 µl blood, a 1.8∶1 promastigote∶leukocyte ratio. Assuming that the number of live opsonized promastigotes in the inoculum is 5%, 0.5 µl of host blood would contain ∼250 live parasites. In the first minutes after infection, granulocytes and monocytes bound parasites at a constant rate, and promastigote binding and ingestion was detectable from 3 min incubation (Fig. 6, insets). At 5 min post-infection, 17.4% of granulocytes bound promastigotes and 8.4% carried TB-unquenched parasites; for monocytes these figures were 10.7% and 2.6%, respectively. At that time, 8.5% of B cells bound promastigotes, but did not internalize them (unpublished data). To illustrate the number of *Leishmania*-infected leukocytes, after 5 min incubation a 0.5 µl blood pool with 5.6×10^6^ leukocytes/ml (66.9% granulocytes, 6.9% monocytes) would have ∼330 granulocytes with bound promastigotes, ∼160 of which would have surface-bound (live) and internalized (live and dead) parasites. In the case of monocytes, there would be ∼20 cells with surface-bound promastigotes and ∼5 cells with surface-bound and internalized parasites.

The early period of *Leishmania* infection was recently addressed in the mouse using intravital two-photon microscopy. Ng *et al.* highlight the role of DDC in invasion, and showed that between 55 and 70% of DDC had ingested parasites by 2 to 3 h post-infection [13]. Host neutrophil depletion before infection did not affect the number of DDC that internalized promastigotes, suggesting that these cells act independently of neutrophils. This apparent lack of neutrophil involvement in infection control could be due to the method of parasite inoculation or to the high intradermal promastigote∶DDC ratio used in these experiments. In any case, the intradermal cell compartment is very different from the hematoma environment; this experiment probably mimics promastigote transmission in a bloodless context, and it is not appropriate to compare it with the blood pool. In another study, Peters *et al.* transmitted infection by intradermal injection of large numbers of promastigotes or with infected flies fed *ad libitum* on restrained mice [17]. Both inoculation methods are likely to have caused dermic hematomas and to have induced substantial neutrophil infiltrate at the injection site. One day post-infection, ∼90% of infected neutrophils harbored live promastigotes; after a week, all leishmanias were inside macrophages and DDC. This change in host cells could be explained by the neutrophil Trojan horse model, which proposes that macrophages are infected when they dispose of apoptotic leukocytes [58]. Peters *et al.* [17] and others [59] nonetheless consider that when infected neutrophils become apoptotic, they release live promastigotes or amastigotes that are phagocytosed by macrophages and DDC (“Trojan rabbit strategy”).

To compare early *Leishmania* infection reactions in mouse and man, we must consider that the earliest host-protective mechanisms, serum complement and professional blood phagocytes, differ in their anti-promastigote activity and in cell proportion in these species. In human blood, the granulocyte∶monocyte ratio is 6–7∶1, whereas in mouse it is from 2–3∶1 [60]. This is important, as these cells are the main parasite targets during infection and thereafter, when they are recruited to the inoculation site. Persistence of neutrophils harboring promastigotes is considered of paramount importance for subsequent disease development [24], [61]–[63].

We anticipate that the size of such a neutrophil reservoir would differ between mouse and man. Human complement is highly cytotoxic to promastigotes, whereas mouse complement is not [28], [64]. After inoculation, we estimate the number of live promastigotes in human blood to be one twentieth of that in mouse, which would considerably reduce the parasite load of infected neutrophils.

**Figure 8:**
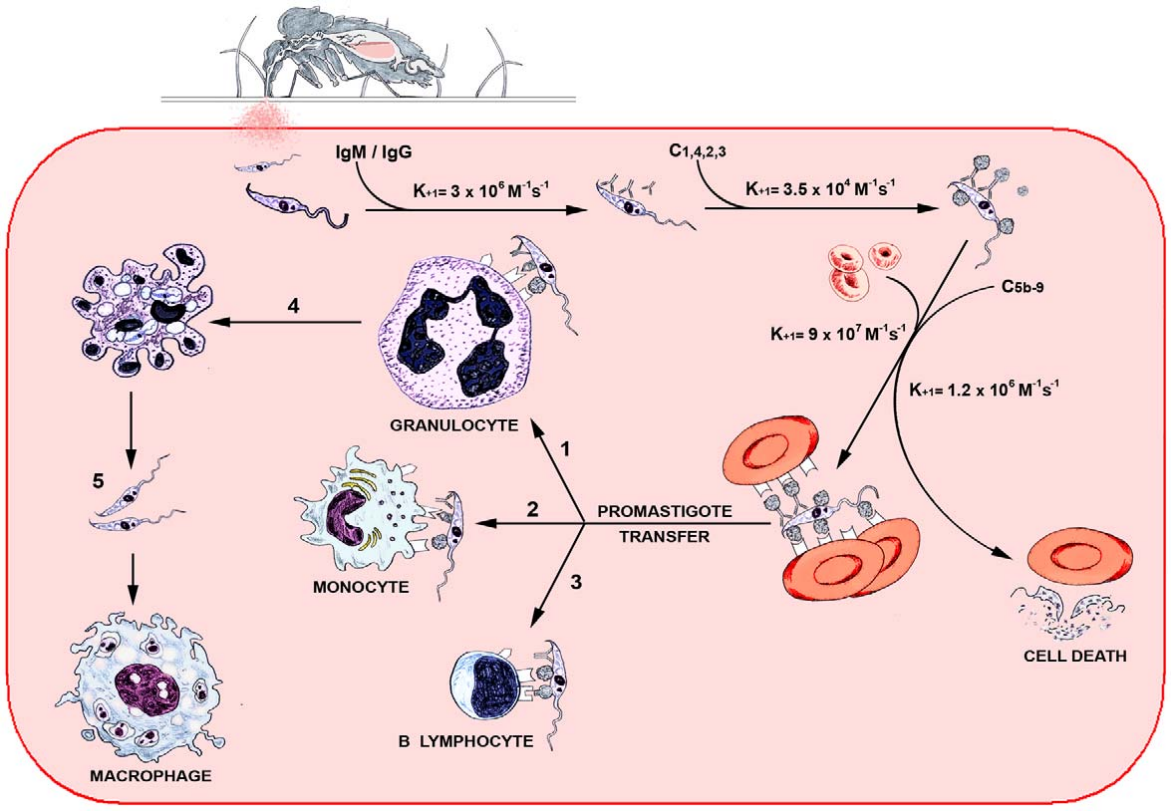
Kinetic model of *Leishmania* infection in the human blood pool. Reaction pathway of promastigotes in host hematoma; k_+1_ constants show the frequency at which the reactions occur. *Leishmania* opsonization by natural antibodies and complement triggers promastigote IA reaction and the complement cytolytic cascade that kills the parasite. The velocity of the IA reaction facilitates parasite transfer to granulocytes (1), monocytes (2) and B lymphocytes (3), causing primary infection in granulocytes and monocytes. Infection can be boosted when parasitized granulocytes that become apoptotic (4) release promastigotes, which are phagocytosed by newly recruited macrophages (5).

Based on these data, we outline a kinetic model of *Leishmania* infection in human blood that incorporates rate constants for promastigote interactions, which measure the speed of these reactions (Fig. 8). At 50 sec post-*Leishmania* inoculation, promastigote opsonization reactions are terminated and complement-dependent parasite killing has not begun, indicating that rapid leukocyte uptake of promastigotes promotes *Leishmania* survival in the host [23], [27]. We suggest that a crucial step in the infection pathway is determined by the high on-rate constant of the *Leishmania* IA reaction. This mechanism competes with complement-mediated parasite lysis by promoting promastigote binding and internalization by granulocytes and monocytes, and binding by B cells (Fig. 8). The substantial number of promastigote-infected leukocytes in the blood pool is probably sufficient to establish host infection. Nevertheless, subsequent macrophage and DDC phagocytosis of promastigotes released from neutrophils, or of apoptotic neutrophils, would boost the parasite load during the early silent phase post-infection.

During natural transmission, sand flies deliver salivary bioactive components to the host, two of which could potentially interfere with early infection mechanisms: saliva and fPPG (PSG). Sand fly saliva inhibits hemostasis and facilitates feeding [65]. Mice inoculated with sand fly salivary gland extracts show exacerbated lesion development, which is associated with early (6 h) increase in type-2 cytokine production and with early (2 to 4 h) expression of macrophage-recruiting chemokines that promote inflammatory cell influx to the injection site [66]–[68].

fPPG inoculated during sand fly bite causes substantial disease exacerbation in mice [6]. fPSG facilitates early parasite establishment by two mechanisms, macrophage recruitment to the infection site and enhancement of alternative macrophage activation, which upregulates arginase activity and promotes amastigote growth [69]. Early leukocyte recruitment to the infection site is detected in vivo from 40 min to 6 h [17] and in vitro after 4 h; the effect of enhanced alternative macrophage activation takes 24–48 h to develop [69]. In mouse, induction of arginase mRNA peaks at day 3 post-stimulation [70] and in man, leukocyte arginase is constitutively expressed only in granulocytes, independently of proinflammatory or anti-inflammatory stimuli [71]. In addition, neither PSG nor sand fly saliva appear to affect macrophage phagocytosis of *L. mexicana* metacyclic promastigotes [69]. We did not have access to PSG material and thus did not examine PSG activity in our system. We therefore cannot assert that PSG does not affect *Leishmania* early infection; nevertheless, the data cited above strongly suggest that PSG and bioactive salivary components exert their effects at a later stage of early infection reactions. Future experiments will help clarify the PSG effect on *Leishmania* ex vivo blood infection.

The most effective innate mechanism against pathogens is said to be denial of access [72]. *Leishmania* has developed an extremely rapid and effective infection strategy, and the prospect of blocking initial promastigote access to a host seems highly improbable. Research efforts should focus on development of therapeutic approaches to prevent *Leishmania* establishment of permanent infection through enhancement of macrophage leishmanicidal mechanisms.

## Supporting Information

Figure S1 Leukocyte binding of promastigotes opsonized with NHS or heat-inactivated serum. Aliquots (50 µl) aliquots of heparinized blood were centrifuged (1200×g, 10 min, 20°C) to separate plasma from cells. Cells were washed twice by centrifugation in PBS and adjusted to the initial blood volume with NHS or heat-inactivated serum (56°C, 60 min; HIS). Reconstituted blood aliquots were incubated (37°C, 5 min) with CMFDA-labeled *L. amazonensis* promastigotes, and leukocyte-promastigote interaction measured as in Fig. 2. Results are expressed as percent (mean ± SEM) of granulocytes (▪) and monocytes (□) that bound promastigotes. Data are derived from three experiments, with blood of different donors.
(0.06 MB TIF)

Figure S2 Time-course of PI uptake by *L. amazonensis* promastigotes incubated with different concentrations of NHS. Single aliquots (0.3 ml) containing *L. amazonensis* promastigotes (2×10^5^), 10 µg/ml PI, and serially diluted (50% to 0.78%) NHS were incubated (37°C) for 1 to 9 min. PI uptake by parasites was measured in real-time flow cytometry (FACSCalibur). Promastigotes were identified and gated by SSC vs. FSC. PI emission was measured in a dot plot of FL-2 (585/42 nm) vs. time (sec) and data were analyzed with CELLQuest software (Becton Dickinson). Time-course of percent promastigote PI uptake in different NHS concentrations: 50% (•), 25% (○), 12.5% (▾), 6.25% (▵), 3.12% (▪), 1.56%(□) or 0.78% (♦).
(0.07 MB TIF)

Figure S3 Percentage of apparently viable *L. amazonensis* promastigotes determined by microscopy examination after incubation in various concentrations of NHS. *L. amazonensis* promastigotes (2×10^7^/ml) were incubated (37°C, 5 min) with pooled NHS serially diluted (1/2) from 50% to 0.78%; the number of apparently live parasites at each serum dilution was counted under a light microscope.
(0.05 MB TIF)

Figure S4Real-time kinetics of PI uptake by *L. amazonensis* promastigotes in NHS, lepirudin- and heparin-treated plasma. Blood samples drawn from healthy donors were immediately centrifuged (1200×g, 10 min, 20°C) to separate plasma from cells, or left to coagulate at 20°C to obtain serum. CMFDA-labeled promastigotes (5×10^5^) were incubated (37°C) in 200 µl aliquots containing 10 µg/ml (final concentration) PI and 50% PBS-diluted NHS or PBS-diluted plasma adjusted to 50 µg/ml final concentration lepirudin (Refludin) or to 0, 10, 12.5, 15, 20, 40 or 80 IU/ml heparin. Parasite killing was measured as PI uptake in real-time flow cytometry (FACSCalibur). Promastigotes were identified and gated by SSC vs. FL-1. PI emission was measured in a dot plot of FL-2 (585/42 nm) vs. time (208 sec). Data were analyzed with CELLQuest software (Becton Dickinson). Time-course of promastigote PI uptake in NHS (+), 50 µg/ml lepirudin-treated plasma (▪), plasma treated with heparin at 10 (○), 12.5 (□), 15 (◊), 20 (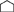), 40 (▵) or 80 (X) IU/ml. A representative experiment is shown.(0.05 MB TIF)

Figure S5 Granulocyte and monocyte binding of *L. amazonensis* promastigotes in lepirudin (50 µg/ml) or heparin (10 IU/ml)-treated blood. Aliquots of treated blood were infected with CMFDA-labeled *L. amazonensis* promastigotes and incubated (37°C) for various times (0–5 min). The reaction was terminated by addition of 2 ml E lysing reagent. After 10 min incubation, 3 ml of sheath fluid was added and tubes were centrifuged (500×g, 5 min); the pellet was washed with 5 ml sheath fluid and resuspended in 200 µl. To calculate the percentage of granulocytes and monocytes that bound parasites, cells were gated by SSC vs. FSC and plotted independently in a secondary plot of SSC vs.FL-1 (green, 530 nm). Results are expressed as the percentage (mean ± SEM) of cells that bound parasites. Data are derived from three experiments, each using blood from a different donor. Blood treated with lepirudin (•) or heparin (○).
(0.08 MB TIF)

Figure S6 Illustrates the percentage of promastigote-binding cells in each leukocyte subpopulation. The analysis was performed as described in Methods for the determination of leukocyte binding of promastigotes. Results are expressed as (mean ± SEM) of five experiments. (▪)*L. amazonensis*, (□)*L. donovani* promastigotes.
(0.11 MB TIF)
