# Supplementary figures and images for: Kinetic Analysis of Ex Vivo Human Blood Infection by Leishmania
Source: PLoS Negl Trop Dis. 2010 Jul 13;4(7):e743. doi: 10.1371/journal.pntd.0000743 (PMC2903471; doi:10.1371/journal.pntd.0000743)

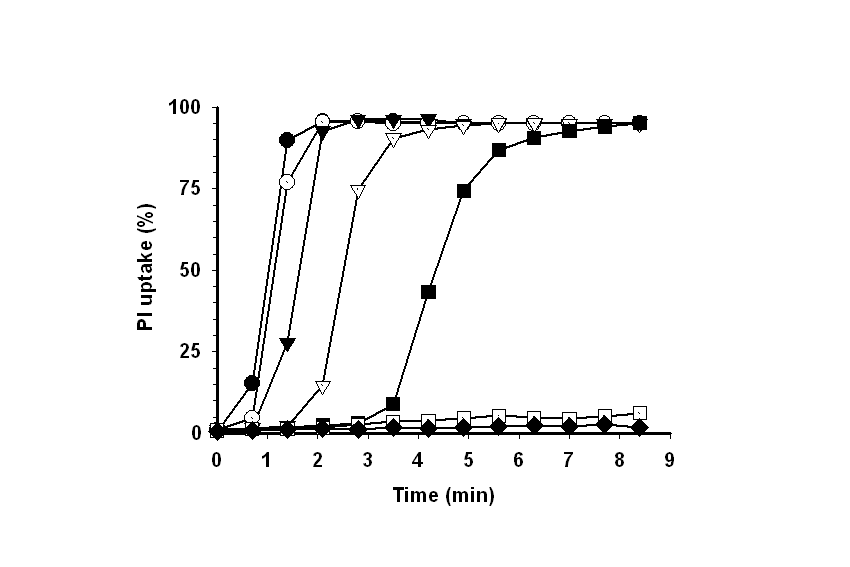

Supplement: Figure S2 — Time-course of PI uptake by L. amazonensis promastigotes incubated with different concentrations of NHS. Single aliquots (0.3 ml) containing L. amazonensis promastigotes (2×10^5^), 10 µg/ml PI, and serially diluted (50% to 0.78%) NHS were incubated (37°C) for 1 to 9 min. PI uptake by parasites was measured in real-time flow cytometry (FACSCalibur). Promastigotes were identified and gated by SSC vs. FSC. PI emission was measured in a dot plot of FL-2 (585/42 nm) vs. time (sec) and data were analyzed with CELLQuest software (Becton Dickinson). Time-course of percent promastigote PI uptake in different NHS concentrations: 50% (•), 25% (○), 12.5% (▾), 6.25% (▵), 3.12% (▪), 1.56%(□) or 0.78% (♦). (0.07 MB TIF) [file pntd.0000743.s002.tif]

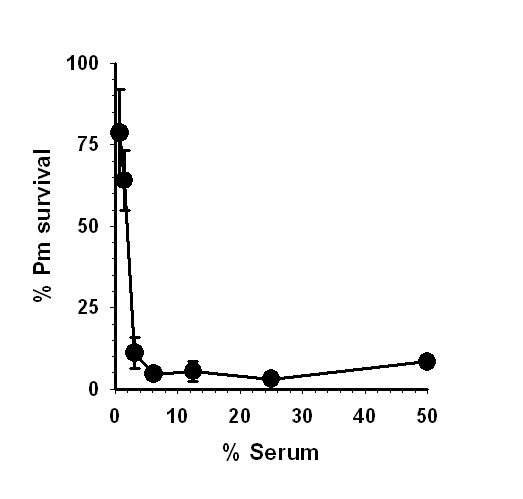

Supplement: Figure S3 — Percentage of apparently viable L. amazonensis promastigotes determined by microscopy examination after incubation in various concentrations of NHS. L. amazonensis promastigotes (2×10^7^/ml) were incubated (37°C, 5 min) with pooled NHS serially diluted (1/2) from 50% to 0.78%; the number of apparently live parasites at each serum dilution was counted under a light microscope. (0.05 MB TIF) [file pntd.0000743.s003.tif]

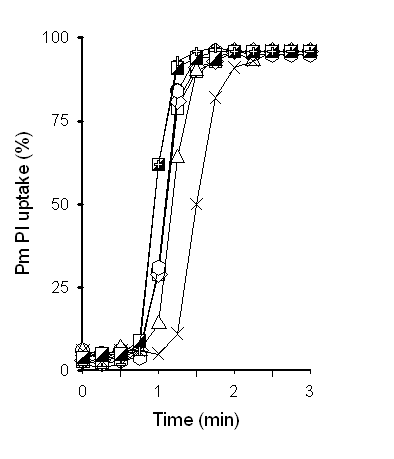

Supplement: Figure S4 — Real-time kinetics of PI uptake by L. amazonensis promastigotes in NHS, lepirudin- and heparin-treated plasma. Blood samples drawn from healthy donors were immediately centrifuged (1200×g, 10 min, 20°C) to separate plasma from cells, or left to coagulate at 20°C to obtain serum. CMFDA-labeled promastigotes (5×10^5^) were incubated (37°C) in 200 µl aliquots containing 10 µg/ml (final concentration) PI and 50% PBS-diluted NHS or PBS-diluted plasma adjusted to 50 µg/ml final concentration lepirudin (Refludin) or to 0, 10, 12.5, 15, 20, 40 or 80 IU/ml heparin. Parasite killing was measured as PI uptake in real-time flow cytometry (FACSCalibur). Promastigotes were identified and gated by SSC vs. FL-1. PI emission was measured in a dot plot of FL-2 (585/42 nm) vs. time (208 sec). Data were analyzed with CELLQuest software (Becton Dickinson). Time-course of promastigote PI uptake in NHS (+), 50 µg/ml lepirudin-treated plasma (▪), plasma treated with heparin at 10 (○), 12.5 (□), 15 (◊), 20 (), 40 (▵) or 80 (X) IU/ml. A representative experiment is shown. (0.05 MB TIF) [file pntd.0000743.s004.tif]

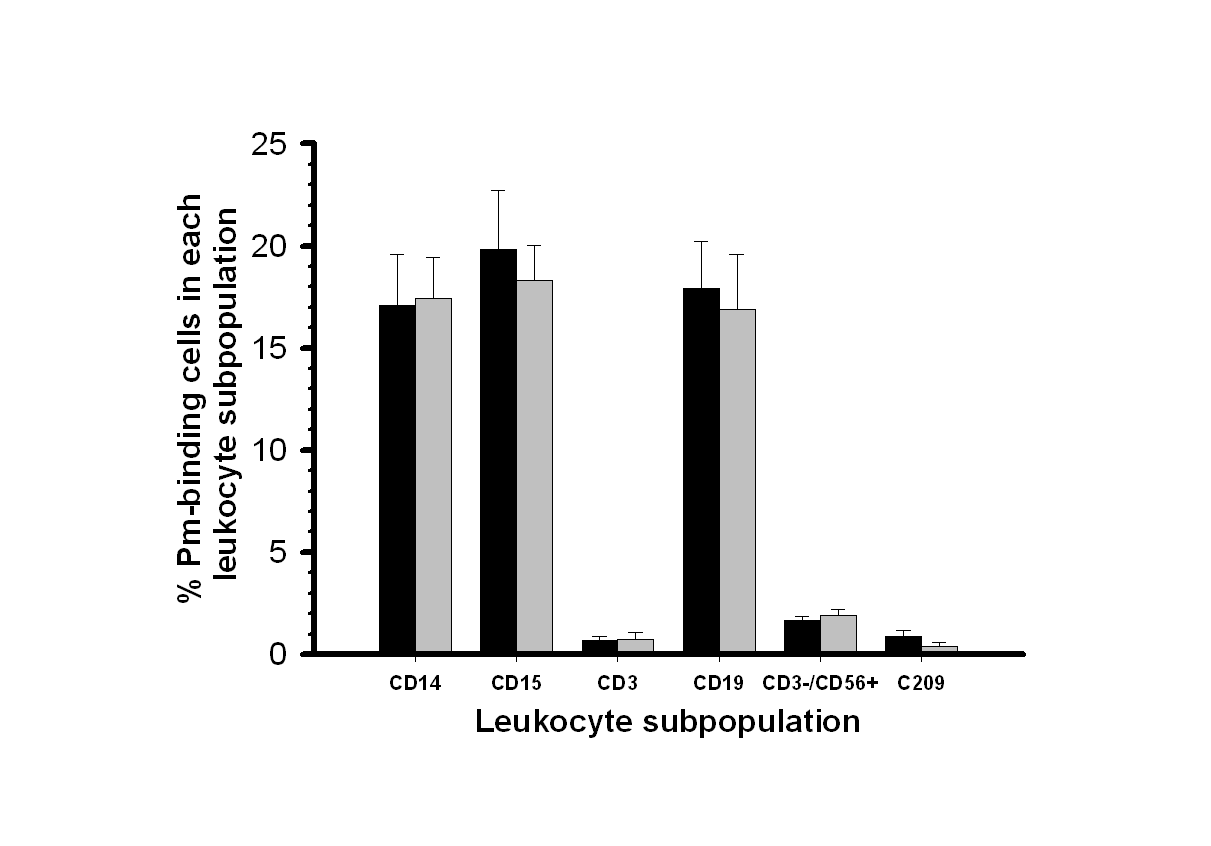

Supplement: Figure S6 — Illustrates the percentage of promastigote-binding cells in each leukocyte subpopulation. The analysis was performed as described in Methods for the determination of leukocyte binding of promastigotes. Results are expressed as (mean ± SEM) of five experiments. (▪)L. amazonensis, (□)L. donovani promastigotes. (0.11 MB TIF) [file pntd.0000743.s006.tif]
